# Determination of cardiac output, shunt-fraction, and active circulatory volume in children with hypoplastic left heart syndrome after the Norwood procedure with RV to PA-shunt.

**DOI:** 10.1038/s41598-026-38858-0

**Published:** 2026-02-04

**Authors:** Anders Aronsson, Theodor Skuli Sigurdsson, Lars Lindberg

**Affiliations:** 1 Institution of Clinical Sciences, Children’s Hospital in Lund, Skane University Hospital, PICU, Lund University, Lasarettsgatan 48, Lund, S-221 85 Sweden; 2 Department of Anaesthesiology and Intensive Care Medicine, National University Hospital of Iceland, 101 Reykjavik, Landspítalinn, Iceland

**Keywords:** Hemodynamics, Hypoplastic Left Heart Syndrome, Neonates, Norwood, Shunt, Single ventricle, Cardiology, Medical research, Physiology

## Abstract

Management of neonates with hypoplastic left heart syndrome following the Norwood procedure seeks to optimize systemic oxygen delivery while maintaining an appropriate distribution between systemic and pulmonary blood flow. The objective of this study was to quantify actively circulating blood volume, total cardiac output, the pulmonary-to-systemic blood flow ratio (Qp/Qs), and vascular resistance indices at fractions of inspired oxygen (FiO2) of 0.21, 0.5, and 0.9 during the early postoperative period. Measurements were obtained in 16 neonates using an ultrasound dilution technique that determines hemodynamic variables from changes in blood ultrasound velocity following injection of isotonic saline (COstatus, Transonic Systems Inc.). An increase in the Qp/Qs ratio was observed with rising FiO2, which was associated with a reduction in systemic blood flow, while pulmonary blood flow remained statistically unchanged across the FiO2 levels studied. This pattern is consistent with the interpretation that pulmonary blood flow in this setting is mainly influenced by the mechanical properties of the right ventricle to pulmonary artery shunt rather than by FiO2 mediated changes in pulmonary vascular tone. Actively circulating volume index was reduced at baseline and decreased further with increasing FiO2 accompanied by an increase in systemic vascular resistance. These findings indicate that the early postoperative hemodynamics in neonates after the Norwood procedure are more responsive to alterations in systemic circulation than to modulation of pulmonary vascular tone. Therapeutic strategies targeting augmentation of circulating blood volume and reduction of systemic afterload warrant further investigation as potential approaches to optimize postoperative management in this population.

**Supplementary Information:**

The online version contains supplementary material available at 10.1038/s41598-026-38858-0.

## Introduction

Patients with a functional single ventricle, such as those with hypoplastic left heart syndrome (HLHS), must undergo a series of complex surgical procedures. These interventions aim to enable the heart to effectively pump blood to both the systemic and the pulmonary circulation. The Norwood procedure is the first stage in this series of operations. It involves the construction of a neoaorta connecting the native aorta to the pulmonary artery, establishing a shunt between the heart and the pulmonary circulation to provide pulmonary blood flow and ensuring unobstructed intracardiac atrial blood flow with an atrial septectomy. This allows the single ventricle to pump blood simultaneously to both the systemic and pulmonary circulation.

The postoperative period is characterized with hemodynamic instability, primarily due to ventricular dysfunction with limited systemic output which has effect on delivered oxygen (DO2), and it is associated with a high risk of morbidity and mortality^1–4^. Postoperative care therefore, focuses on optimizing ventricular function and achieving a balanced distribution of pulmonary (Qp) and systemic (Qs) blood flow^3,5^.

To guide clinical management, oxygen consumption (VO2) and estimation of Qp/Qs ratio by using the modified Fick’s equation have been used^3,6–8^. However, this approach can be misleading as the application of the Fick method requires precise measurement of VO2 and arteriovenous oxygen content difference^9^. In paediatric populations, VO2 is highly variable and strongly influenced by age, growth, temperature, sedation, agitation, and disease state^10^. Concurrent pulmonary venous desaturation and low systemic venous saturation may result in an erroneous underestimation of the Qp/Qs ratio^5^. Given that Qs is a key determinant of end-organ perfusion and DO2, such underestimation can result in inadvertent systemic hypoperfusion. Moreover, hemodynamic stability depends not only on Qs but also on the circulating blood volume and total cardiac output (Q), parameters that are challenging to assess accurately using physical examination, arterial blood pressure, central venous pressure, or blood gas analysis^11^.

Reliable methods for quantifying hemodynamic variables following the Norwood procedure remain limited, resulting in an incomplete characterization of circulatory dynamics in single-ventricle physiology. Accurate hemodynamic assessment is particularly challenging in neonates because of their low body weight, limited vascular access, and physiologic vulnerability in the immediate postoperative period.

A technology that uses ultrasound sensors to measure blood ultrasound velocity before and after injection of body temperature isotonic saline has been developed. The changes in blood ultrasound velocity produces blood dilution curves that assess blood flow based on the behaviour of the injectate and thereby measures hemodynamic parameters^12–14^. It has been validated for detection of intracardiac shunts in experimental animals, neonates and children with shunt dependent circulation and found to have high sensitivity and specificity^15–17^. This technology makes it possible to evaluate actively circulating volume (ACV), stroke volume (SV), total cardiac output (CO) and the Qp/Qs ratio^17,18^. Measurement of ACV are of particular interest in these vulnerable patients, as it directly have impact on cardiac preload and, consequently, cardiac function^19^.

The aim of this study was to determine these hemodynamic variables and evaluate their response to varying FiO2. Oxygen acts as a pulmonary vasodilator^20^, rapidly dilating pulmonary arterioles and increasing Qp in congenital heart diseases, both with and without shunt physiology^20–23^. This vasodilatory response is typically manifested within minutes^23^. Although in HLHS after the Norwood procedure prior research studies indicate that the shunt restricts Qp effectively and protects from FiO2 caused pulmonary vasomotion. We therefore, hypothesized that increasing FiO2 would not affect the measured hemodynamic variables.

## Materials and methods

Patients scheduled for a Norwood procedure were eligible for this study. Inclusion criteria were single ventricle physiology and surgery within the first week after birth and parental consent. Appropriate preoperative stabilization preceded the surgical procedure. Patients underwent surgery with arch reconstruction, placement of a RV-PA shunt (6 mm), and creating an unobstructed atrial communication.

Sixteen neonates were included in this prospective observational study. There were 10 boys and 6 girls. Survival rate was 100% at three years follow-up. The study was approved by the Ethics Committee of Lund University, Lund, Sweden (Dnr 2013/636 and Dnr 2016/514). Informed consent from the parents of the children was obtained. The patients were managed according to relevant guidelines and regulations.

Weight and ages at surgery, sternal closure and hemodynamic measurement are presented in Table 1.

**Table 1:** Weight and ages at surgery, sternal closure and measurement.

Variables	Median	Range
Weight (kg)	3.4	2.9–4.0
Age at surgery (days)	4	1–7
Age at sternal closure (days)	8	6–11
Age at hemodynamic measurement (days)	10	7–12

Invasive arterial blood pressure (measured from the arterial line positioned in the arteria tibialis posterior), central venous pressure (CVP) (measured from the distal end of the central venous line), and heart rate (HR) were recorded and arterial and venous blood gases collected. Hemodynamic measurements were made by using body temperature isotonic saline blood dilution detected by ultrasound sensors (COstatus monitor device, Transonic Systems Inc., Ithaca, NY, USA)^24^. The saline was injected into the central venous line (distal lumen of the central venous catheter) of an arterial-venous (AV) loop connected to the circulatory system of the neonate (between the arterial line in the right arteria radialis and the central venous catheter in the right jugularis interna). This injection produced a dilutional curve that was used to calculate total CO using a modified version of the Stewart-Hamilton equation^4,25^. It is possible to distinguish between the first passage (Qs) and the second passage of blood (recirculation – Qp), since the technology uses an external roller pump which results in a stable blood flow of 10–12 ml/min in the extra-corporeal AV loop between existing arterial and central venous lines, to which the ultrasound detectors are connected. A rapid rise in the ascending part and/or a delay in the declining part of the dilution curve, detected by the ultrasound sensor on the arterial limb, occur in children with single-ventricle due to the mixing of blood between the pulmonary and systemic circulations. The software of the device uses the undistorted part of the dilution curve to create an imaginary plausible area under the curve (AUC) without signs of recirculation, representing measured Qs. This AUC can be compared with the actual AUC, representing total CO, which also contains the recirculated blood. This makes it possible to calculate the Qp/Qs ratio. The method has been validated in small children with shunts^12,16,25^.

Total CO, Qs, and ACV were quantified by the software and Qp/Qs ratio, SV, SVR ((MAP - CVP)/Qs) and tPVR ((MAP – CVP)/Qp) were calculated^8,26^. Total pulmonary vascular resistance included the resistances across the RV-PA shunt and the pulmonary vasculature as the individual resistances are added in serial connections. Appropriate hemodynamic variables were indexed to weight (CI, SVI, ACVI, tPVRI, and SVRI), since the body surface area (BSA) in children less than 15 kg are non-linear related to body weight^25^.

Subjects were studied in the pediatric intensive care unit (PICU). During the study they were lightly sedated and mechanically ventilated. All were normothermic (36.4–37.4 °C) and had their sternum closed. Sedation and analgesia were initiated during the operation with dexmedetomidine (0.3–1.4 mcg/kg/hour), ketobemidone (18–30 mcg/kg/hour) and this was continued during the postoperative period. Propofol (1–3 mg/kg/hour) was added as a complement to the established sedation regime during the measurements. All neonates were on inotropic support with a constant dose of milrinone (0.5 mcg/kg/minute), which had been initiated during the operation. Four neonates were also on a constant infusion of low dose of norepinephrine (0.04–0.09 mcg/kg/minute). None of the continuous infusions were changed during the measurement sessions. Ventilatory support was maintained on ServoI (Maquet) ventilators with pressure regulated volume control, tidal volumes of 6–7 ml/kg, and a PEEP of 6–9 cmH2O.

Hemodynamic measurements were obtained at FiO2 of 0.21, 0.50, and 0.90. The protocol began at an FiO2 of 0.21, which was subsequently increased in stepwise increments of 0.10 while continuously monitoring the neonate’s circulatory status. Each FiO2 increment was maintained for a minimum of five minutes to allow hemodynamic stabilization before proceeding to the next level. Once the inspiratory and expiratory oxygen fractions were confirmed to be stable at the target FiO2 values (0.21, 0.50, and 0.90) using a Deltatrac metabolic monitor (Datex-Ohmeda), and after an additional stabilization period of at least five minutes, arterial blood gases were obtained and hemodynamic measurements were performed. Blood gas analysis was performed with the ABL800 Flex Radiometer (Radiometer AS, Brönshöj, Denmark). All children had two arterial lines (right radial and tibialis posterior arteries) and a 3-lumen central line placed in the right internal jugular vein. Each measurement session consisted of at least three consecutive repeated measurements with body temperature physiological saline boluses (1 ml/kg) spaced 60 to 120 s apart. If the patient moved or if there were an unreliable curve indicated by the COstatus software, additional injections were performed, never exceeding five injections. The total number of injections was 176. The COstatus software failed to give data in five of these. Of the remaining 171, 25 injections failed to give an ACV value but gave all other hemodynamic data. Failure to obtain an ACV value was in all cases due to movements of the lightly sedated patient. All of the measurements were obtained by the authors.

Statistical analysis was performed using SPSS version 29.0.2.0 (IBM SPSS Statistics). No statistical power analysis was conducted before the study as it was designed as convenience sampling.

Shapiro—Wilk test indicates that half of the measurements deviate significantly from a normal distribution. Data were therefore expressed as median and interquartile range (IQR). The non-parametric Friedmans analysis of variance (ANOVA) for repeated dependent measurements was used to detect significant differences in the variables between the measurements at 0.21, 0.5 and 0.9. A p-level of less than 0.05 was considered as significant after correction for multiple comparisons. Within-subject coefficient of variation (WCV) was calculated as the ratio of the within subject standard deviation to the mean. Confidence interval was calculated as sample mean with z value of 1.96*, (95% CI = mean ± 1.96 * SD/√N). (Supplementary Table S1)

The availability of COstatus device has been globally discontinued since 2017 and the AV loops since 2022 by decision of the company.

## Results

**Fig. 1a:**
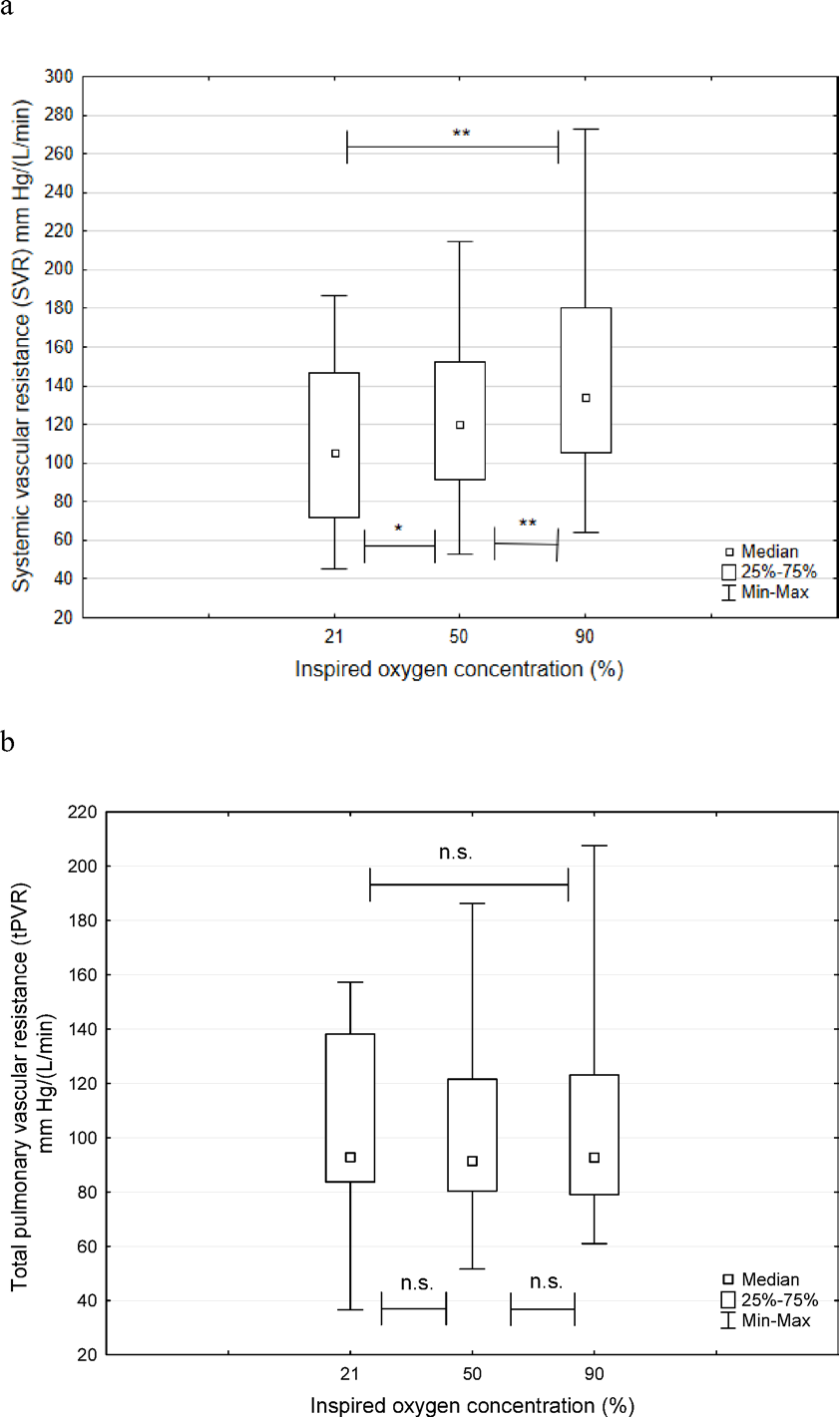
and 1**b** Systemic vascular resistance (SVR), Total pulmonary vascular resistance (tPVR), shown at different levels of inspired oxygen concentration (21, 50 and 90%), values are presented as median, IQR and Min-Max.

When FiO2 was increased from 0.21 to 0.9 the hemodynamics responded with a decrease in Qs, while Qp remained statistically unchanged under the conditions studied. The increase in FiO2 did not affect tPVR or Qp statistically (Table 2; Fig. 1a). Therefore, the observed increase in the Qp/Qs ratio at higher FiO2 levels was solely due to a reduction in Qs (Table 2).

**Table 2:** Systemic blood flow (Qs), pulmonary blood flow (Qp), and shuntfraction (Qp/Qs). Values are median and range. Friedmans analysis of variance (ANOVA) for repeated dependent measurements.

Variables	FiO2 0.21	FiO2 0.50	FiO2 0.9	*p*-value (0.21 vs. 0.5)	*p*-value (0.21 vs. 0.9)
Qs (L/min)	0.41 (0.22–0.66)	0.35 (0.16–0.55)	0.32 (0.13–0.47)	*p* < 0.001	*p* < 0.001
Qp (L/min)	0.43 (0.20–0.77)	0.45 (0.22–0.84)	0.45 (0.18–0.62)	ns.	ns.
Qp/Qs	1.19 (0.30–2.59)	1.44 (0.41–2.91)	1.62 (0.62–4.23)	*p* < 0.001	*p* < 0.001

Total CO, expressed as the cardiac index (CI), decreased with increasing FiO2. However, the stroke volume index (SVI) of the single ventricle remained unchanged, indicating preserved cardiac function. The decrease in CI at higher FiO2 was attributed to an increase in systemic vascular resistance (SVR) and a decrease in heart rate (HR). (Table 3; Fig. 1b).

**Table 3:** Mean arterial pressure (MAP), central venous pressure (CVP), heart rate (HR), cardiac index (CI), actively Circulating volume indexed (ACVI), stroke volume index (SVI) and hemoglobin. Values are median and range. Friedmans analysis of variance (ANOVA) for repeated dependent measurements.

Variables	FiO2 0.21	FiO2 0.50	FiO2 0.9	*p*-value (0.21–0.5)	*p*-value (0.21–0.9)	*p*-value (0.5–0.9)
MAP (mmHg)	47 (45–51)	47 (45–50)	49 (45–53)	ns.	ns.	ns.
CVP (mmHg)	7 (6–9)	7 (6–8)	7 (6–8)	ns.	ns.	ns.
HR (beats/min)	130 (128–146)	128 (126–144)	127 (117–141)	*p* < 0.01	*p* < 0.001	*p* < 0.01
ACVI (ml/kg)	54 (38–77)	49 (30–65)	45 (25–60)	*p* < 0.05	*p* < 0.001	*p* < 0.05
CI (L/min/kg)	0.24 (0.15–0.31)	0.23 (0.14–0.32)	0.22 (0.14–0.27)	*p* < 0.05	*p* < 0.01	ns.
SVI (ml/kg)	1.84 (1.08–2.73)	1.76 (1.08–2.49)	2.05 (0.86–3.75)	ns.	ns.	ns.
Hemoglobin (g/L)	14.7 (14.2–15.5)	14.2 (13.4–15.0)	13.9 (13.3–14.8)	*p* < 0.01	*p* < 0.01	ns.

Mean arterial pressure (MAP) and CVP did not change significantly across the different levels of FiO2 (Table 3).

The actively circulating volume index (ACVI) of 54 ml/kg (70 ±15 ml/kg – values in children with biventricular physiology after cardiac surgery) decreased with increasing FiO2 (Table 3).

Both arterial oxygen saturation (SaO2) and venous oxygen saturation (SvO2) increased with higher FiO2, while the arteriovenous saturation difference (SaO2 – SvO2) remained unchanged (Table 4).

**Table 4:** Arterial blood saturation (SaO2), venous blood saturation (SvO2), and the arteriovenous saturation difference (saO2-SvO2). Values are median and range. Friedmans analysis of variance (ANOVA) for repeated dependent measurements.

Variables	FiO2 0.21	FiO2 0.50	FiO2 0.9	*p*-value (0.21 vs. 0.5)	*p*-value (0.21 vs. 0.9)	*p*-value (0.5 vs. 0.9)
SaO2 (%)	76 (72–79)	89 (83–90)	94 (90–96)	*p* < 0.001	*p* < 0.001	*p* < 0.01
SvO2(%)	55 (42–63)	69 (58–73)	73 (62–78)	*p* < 0.001	*p* < 0.001	*p* < 0.01
SaO2-SvO2 (%)	18 (15–27)	20 (17–25)	23 (16–29)	ns.	ns.	ns.

## Discussion

Our results offer several observations that contribute to the understanding of immediate postoperative hemodynamic physiology in neonates with single-ventricle circulation following the Norwood procedure with a RV–PA shunt. Several findings warrant particular consideration.

We observed that the Qp/Qs ratio increased with higher FiO2, which was primarily associated with a reduction in Qs. This pattern is consistent with the interpretation that, in this early postoperative period, Qp is largely determined by the mechanical properties of the RV–PA shunt rather than by dynamic changes in pulmonary vascular tone. Given that the shunt functions primarily as a passive conduit without intrinsic regulatory capacity, changes in pulmonary vascular resistance may have a limited effect on Qp during this period. This observation suggests that the physiological impact of pharmacologic pulmonary vasodilation could be modest in this specific clinical context.

While the concept of actively balancing pulmonary and systemic blood flow is frequently emphasized in the management of single-ventricle physiology, our findings suggest that, under the conditions studied, variations in the Qp/Qs ratio predominantly reflect changes in Qs rather than reciprocal regulation of Qp. Notably, increasing FiO2 did not appear to augment Qp, implying that any oxygen-induced reduction in pulmonary vascular tone may have limited influence on Qp in the presence of a mechanically fixed restrictive shunt. Furthermore, the observed stability of the driving pressure across the pulmonary circulation supports this interpretation. These findings are in agreement with previous reports, including those by Naito et al.^27^ which suggest that resistance within the RV-PA shunt may represent a dominant determinant of Qp during this postoperative phase.

It is commonly assumed that elevated oxygen levels induce dilation of precapillary pulmonary arterioles, thereby reducing pulmonary vascular tone. In the context of a mechanically regulated Qp and stable driving pressure, such vasodilation may not substantially alter tPVR. Instead, it might redistribute pressure gradients toward the distal pulmonary vasculature, potentially increasing capillary hydrostatic pressure. This mechanism could, in turn, favor transcapillary fluid filtration and increased interstitial lung water, which may raise diffusion resistance across the alveolar–capillary membrane. While speculative, this hypothesis raises the possibility that prolonged exposure to high inspired oxygen concentrations and pulmonary vasodilators could adversely affect pulmonary gas exchange in this patient population.

In addition, increasing FiO2 was associated with a reduction in Qs as measured by COstatus. Given that MAP and CVP remained unchanged, this observation is compatible with the presence of increased SVR. Although this vasoconstrictive response may not be readily apparent clinically, it has the potential to reduce systemic oxygen delivery (Qs × CaO₂), particularly at FiO2 exceeding 0.5. These findings highlight the need for careful consideration of oxygen therapy in the early postoperative management of neonates following the Norwood procedure.

Our results agree with the findings of others that neither respiratory alkalosis nor short episodes of increased inspiratory levels of oxygen causes recognizable changes in MAP, CVP, nor Qp in this patient category^28,29^. It also agrees with the finding that Qp/Qs was insensitive to manipulation of the pulmonary vascular tone, and that Qp was limited by the RV-PA shunt^30,31^. A study evaluating the response on the tPVR to different levels of FiO2 supports that the shunt limits Qp^30^. In addition, increases in pCO2 induced direct effects on the systemic perfusion rather than on pulmonary blood flow, also indicating small changes in tPVR and Qp in neonates after the Norwood procedure^32^. This may explain why assumed oxygen consumption has been difficult to use as a determinant of CO and results in large errors in the calculated values of tPVR^33,34^. It may also explain the poor correlation between Qp and DO2, SvO2, Sa-vO2, and oxygen excess factor (SaO2/Sa-vO2)^33^.

Another observation of interest is that these children have a significant low ACVI, amounting to 25% of the ACVI of children scheduled for correction of shunts with biventricular circulation^25^. This finding occurred despite our policy of a liberal use of blood products and Albumin 5%. Observation of a low ACVI, which contributed to a systemic hypoperfusion, occurred while the SVR remained high to maintain MAP and CVP. This relative hypovolemia likely reflects the unique loading conditions imposed by the parallel circulation and the postoperative systemic response. Notably, we observed a further reduction in ACVI at higher inspired oxygen fractions, which may result from oxygen-induced alterations in vascular tone or shifts in intravascular fluid distribution.

This study provides insights to the vulnerability and challenges that may complicate the management of these children. A decrease in circulatory blood volume causes systemic vasoconstriction to maintain MAP at the expenses of lower Qs. Since tPVR is mainly regulated by the shunt and MAP is unchanged or minimally affected, Qp will be less affected than Qs, by hypovolemia and hypoperfusion. Qp receives a larger fraction of the total Q. A slight decrease in circulatory blood volume, such as can occur during inflammatory responses or infections may imply that a larger fraction of the existing total blood volume is prioritized to the pulmonary circulation, thereby compromising systemic perfusion even more. A low ACV might stimulate the renin-angiotensin-aldosterone-system activity to maintain arterial blood pressure by increasing SVR and the sympathetic nervous system activity to maintain preload by increasing venous tonus opposing the need of afterload reduction which has been found to be beneficial^35^.

Neonates with single ventricle and compensated hypovolemia may be especially vulnerable to afterload reduction with a decrease in diastolic blood pressure and accordingly affecting regional tissue oxygenation^36^. This may be more pronounced in children with lower weight at surgery and those being anemic, who have a lower total blood volume. It seems judicious to balance afterload reduction with an expansion of the circulatory volume in order to compensate for the decrease in SVR.

The postoperative course after Norwood procedure is also characterized by dysoxygenation caused by pulmonary gas exchange abnormalities (ventilation-perfusion mismatch)^30^. The improvement of SaO2 without change in Qp, when FiO2 increases, indicated that the desaturation is mainly caused by an increased diffusion resistance to oxygen across the alveolar-to-capillary membrane and not due to change in Qp. SaO2 has also been shown to be a poor predictor of Qp/Qs^30,37–39^.

The major restriction of Qp occurs in the RV-PA shunt, making pulmonary vascular tone in itself relatively unimportant as a regulator of Qp^27^. The surgical decision of the size of the shunt is therfore more important than later attempts trying to regulate Qp by vasodilators^28,31,40,41^.

Our findings also indicate that SaO2 is mainly dependent on SvO2 which in turn ultimately depends on Qs. Qs is dependent on circulatory blood volume and SVR. This may explain why DO2 is most closely correlated with SVR and Hb and not SaO2^8^.

It also explains why SaO2 increases temporarily when the neonates, during the first months after surgery grow, the RV-PA shunt becomes relatively more restrictive, and the blood flow ratio diverge against Qs rather than Qp. It increases DO2 and SvO2 initially, without significantly influence the need of Qp to maintain a sufficient oxygenation of the pulmonary venous blood. However, after a few months the shunt becomes restricted, the child becomes more hypoxic, and then the Glenn anastomosis is needed.

An interesting finding was also the increase of SVR at higher FiO2. This has been demonstrated in healthy subjects and in patients with heart failure but not in sedated neonates with single ventricle physiology^42^. A slightly higher FiO2, below 0.4 might be beneficial to overcome the ventilation-perfusion mismatch, but FiO2 at 0.5 and above might cause vasoconstriction counteracting afterload reduction and opposes the aim of the management to improve Qs and DO2. Hyperoxia has been demonstrated to have detrimental effects in acyanotic children^10,43^.

This study has several limitations that should be considered when interpreting the findings. The cohort size was relatively small, and observations were confined to the immediate postoperative period. During the study, all neonates were lightly sedated with propofol and dexmedetomidine and remained mechanically ventilated, which may have influenced hemodynamic conditions and limits the generalizability of the results to other clinical settings.

Hemodynamic measurements were obtained at three predefined levels of FiO2. In accordance with institutional practice, milrinone therapy was maintained throughout the study period to support ventricular function, thereby reducing variability in myocardial performance but potentially limiting extrapolation to conditions in which inotropic or vasodilatory support differs. As expected in a heterogeneous population of neonates with single-ventricle physiology, the hemodynamic variables demonstrated substantial interindividual variability.

Pulmonary blood flow increased by a mean of 20 mL/min between subjects when FiO2 was increased from 0.21 to 0.5. This increase was slightly greater than the within-subject coefficient of variation (WCV) for Qp of 11.8%, corresponding to a within-subject variability of 11 mL/min. However, this change was not significant in the nonparametric analysis of variance for repeated measures, indicating no consistent within-subject change in Qp. The small, nonsignificant increase in Qp observed between subjects with increasing FiO2 may reflect an effect of oxygen on pulmonary vascular vasomotion, but no clear trend was evident that this affected Qp. Because calculated total pulmonary vascular resistance (tPVR) did not change significantly either within or between subjects, it suggests that the mechanically fixed shunt was the primary determinant of Qp.

Venous blood samples were obtained from the superior vena cava and therefore may not fully represent true mixed venous oxygen saturation. Estimation of the pulmonary-to-systemic blood flow ratio relied on the mathematical algorithms incorporated in the COstatus system. Although dilution curves deemed ambiguous were excluded from analysis, the method is known to exhibit a degree of inaccuracy and imprecision in shunt ratio estimation. Moreover, while the technology has been validated in pediatric patients with large intracardiac and extracardiac shunts, it has not been specifically validated in neonates with single-ventricle physiology.

Ideally, additional validation of the COstatus system in this patient population would be desirable. However, direct measurement of Qp and Qs in single-ventricle physiology during cardiac catheterization is technically challenging, and accurate determination of oxygen consumption is particularly difficult in the presence of high inspired oxygen concentrations. As a result, conducting rigorous validation studies in the immediate postoperative setting is inherently complex. Although cardiac magnetic resonance imaging could theoretically be considered for validation purposes, its application in this context is constrained by the fragility of the postoperative circulation and practical limitations related to monitoring, support equipment, and metallic components near the scanner. Consequently, further validation was not feasible within the scope of the present study. The withdrawal of the COstatus from the market makes independent replication of our study not possible to perform at the moment.

Our findings suggest that postoperative management following the Norwood procedure may place particular emphasis on strategies aimed at supporting and maximizing Qs, including optimization of intravascular volume status and modulation of systemic afterload. Within the conditions studied, Qp appeared to be largely constrained by the mechanical properties of the RV-PA shunt, which may limit the physiological impact of pharmacologic pulmonary vasodilation in the immediate postoperative period. In this context, pulmonary vasodilator therapy may have a very limited effect on Qp and could potentially be associated with increased capillary filtration pressure, thereby favoring interstitial fluid accumulation, although this possibility remains speculative.

In addition, our observations indicate that modest increases in the FiO2 were not associated with overt hemodynamic instability in this cohort. Such an approach may contribute to improved oxygen transfer by reducing diffusion resistance across the alveolar–capillary membrane; however, the balance between potential benefits and unintended effects warrants further investigation.

## Conclusions

Consistent with prior indirect evidence, our hemodynamic assessment suggests that pulmonary blood flow in the immediate postoperative period after the Norwood procedure may be relatively constrained and influenced by the mechanical properties of the RV–PA shunt. Our findings also indicate that effective circulating blood volume in neonates with single-ventricle physiology may be reduced compared with biventricular circulation. Although these observations should be interpreted cautiously given the limitations of hemodynamic assessment in this population, they highlight the delicate balance between systemic and pulmonary blood flow, support an individualized approach to postoperative volume and oxygen management, and underscore the need for further studies to clarify underlying mechanisms and clinical significance.

## Supplementary Information

Below is the link to the electronic supplementary material.

Supplementary Material 1

## Data Availability

The datasets generated during and/or analysed during the current study are available from the corresponding author on reasonable request.

## Abbreviations

ACV: Actively Circulating Volume
ACVI: Actively Circulating Volume Indexed
AUC: Area Under The Curve
CI: Cardiac Index by Weight
CO: Cardiac Output
CVP: Central Venous Pressure
DO2: Delivered Oxygen
FiO2: Fraction of Inspired Oxygen
HLHS: Hypoplastic Left Heart Syndrome
HR: Heart Rate
MAP: Mean Arterial Pressure
PA: Pulmonary Artery
PVR: Pulmonary Vascular Resistance
Q: Cardiac Output
Qp: Pulmonary Blood Flow
Qs: Systemic Blood Flow
Qp/Qs: Shunt fraction
RV: Right Ventricle
RV-PA: Right Ventricle to Pulmonary Artery
SV: Stroke Volume
SVR: Systemic Vascular Resistance
tPVR: Total Pulmonary Vascular Resistance
VO2: Oxygen Consumption
