# Supplementary material for: Determination of cardiac output, shunt-fraction, and active circulatory volume in children with hypoplastic left heart syndrome after the Norwood procedure with RV to PA-shunt
Source: Sci Rep. 2026 Feb 4;16:4748. doi: 10.1038/s41598-026-38858-0 (PMC12873426; doi:10.1038/s41598-026-38858-0)
Supplement: Supplementary file 1 — Supplementary Material 1 [file 41598_2026_38858_MOESM1_ESM.docx]

|  | WCV | Mean | CI 95% | CI min | CI max |
| --- | --- | --- | --- | --- | --- |
| CO | 0,070 | 0.792 | 0.007 | 0.786 | 0.798 |
| Qs | 0.064 | 0.361 | 0.003 | 0.359 | 0.364 |
| Qp | 0.118 | 0.431 | 0.007 | 0.426 | 0.437 |
| Qp/Qs | 0.145 | 1.386 | 0.024 | 1.366 | 1.407 |
| ACVi | 0.102 | 48.03 | 0.583 | 47.54 | 48.52 |

Supplementary Table S1 Within-subject coefficient of variation (WCV), Mean and confidence interval (CI) with min-max.
